# Managing solar retinopathy with suprachoroidal triamcinolone acetonide injection in a young girl: a case report

**DOI:** 10.1186/s13256-021-03162-0

**Published:** 2021-12-02

**Authors:** Ameen Marashi, Marwa Baba, Aya Zazo

**Affiliations:** 1 Retina Specialist at Marashi Eye Clinic, Aleppo, Syria; 2 Ophthalmic Resident at Aleppo Eye Surgical Hospital, Aleppo, Syria; 3 Faculty of Medicine, University of Aleppo, Alhamadaniah Aleppo, Syria

**Keywords:** Solar retinopathy, Suprachoroidal, Injection, Triamcinolone acetonide, Efficacy, Visual loss

## Abstract

**Background:**

Solar retinopathy is a disease that causes photochemical toxicity in the retinal fovea tissues, leading to an acute decrease of vision.

**Case presentation:**

This case report is an interventional case of an asymptomatic 17-year-old Caucasian female with a history of suddenly decreased vision due to solar retinopathy. The patient was managed with a custom-made needle injection of triamcinolone acetonide in the suprachoroidal space. Four months post suprachoroidal injection showed an anatomical and functional improvement in the ellipsoid zone layer through optical coherence tomography signal reappearance. In addition, the best-corrected visual acuity had improved from 0.1 to 1.0 on the Snellen chart with the disappearance of the scotoma. However, there was a mild increase in intraocular pressure after this procedure, controlled with topical hypertensive eye drops.

**Conclusion:**

Suprachoroidal triamcinolone acetonide injection using a custom-made needle showed both functional and anatomical improvement of macular changes post-solar retinopathy, with acceptable safety outcomes in a young female.

## Background

Solar retinopathy is an injury of retinal tissues owing to photochemical toxicity in the fovea, induced from solar or eclipse gazing, causing a reduction of central vision [1]. Patients who suffer from solar retinopathy may experience central scotoma, photophobia, and chromatopsia [2]. In addition, the absorbed sunlight to the melanin located in the retinal pigment epithelium (RPE) cells induces thermal and photochemical injury to the photoreceptors and RPE cells [3].

In our case, we report a young female suffering from solar retinopathy who presented to an ophthalmic clinic after central visual loss for 4 weeks.

## Case presentation

**Fig. 1:**
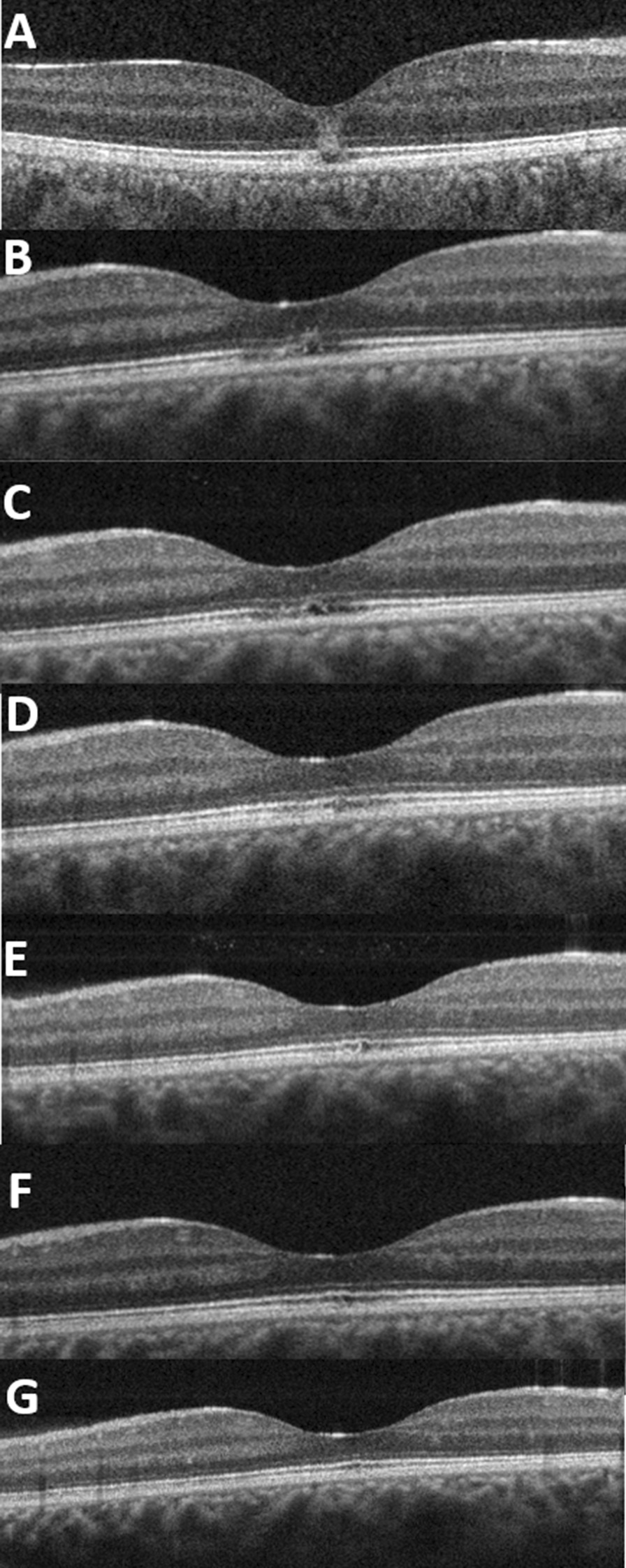
**A** A discontinuity of the ellipsoid and interdigitation zones, with an increased overlying tissue reflectivity due to inflammatory reaction. **B** Four weeks post initial presentation and before suprachoroidal injection, OCT cross-section shows discontinuity features of the ellipsoid zone and interdigitation zone, with mild disruption of the external limiting membrane with the resolution of most overlying tissue reaction. **C** One week post-suprachoroidal injection presents completely resolved tissue reaction with the discontinuity of the interdigitation zone and signal reappearance of the ellipsoid zone, but it looks disrupted. **D** One month post-suprachoroidal injection shows reappearance of the ellipsoid zone’s signal, with disturbance and discontinuity of interdigitation zone. **E** Two months post-injection shows mild disturbance of the ellipsoid zone, with interdigitation zone signal reappearance and disturbance. **F** Three months post-injection presents only disturbance in the interdigitation zone. **G** Four months post-injection presents a normal OCT.

A 17-year-old Caucasian female presented to a private ophthalmic clinic with a complaint of decreased vision for 6 weeks because the patient gazed at the sun to take a “selfie.” The patient never smoked, nor was she a regular user of alcohol. The patient was healthy and reported no significant prior medical or family history. One month ago, the patient was referred to a hospital where her best-corrected visual acuity (BCVA) was 0.1. The optical coherence tomography (OCT) scans showed an ellipsoid zone and interdigitation zone disruption, with an increased hyperreflectivity of the overlying tissues due to the inflammation process (Fig. 1A). However, no fundus images were available from her first clinical examination in the hospital.

When the patient visited the private clinic, the BCVA of her right eye was 0.4 (with refraction −0.75 × 173), whereas the BCVA of her left eye was 1.0 (with refraction +1.75/−1.00 × 9). Clinical examination showed unremarkable features in the anterior segment of both eyes. Fundus examination showed a dull foveal reflex in the right eye, while the left eye was normal. OCT scans of the right eye showed ellipsoid zone and interdigitation zone disruption with hyperreflectivity of overlying tissues (Fig. 1B). However, the lesion was less intense than the acute phase. OCT scans of the left eye were normal.

After that, a consultation with the patient’s parents to do a suprachoroidal injection of triamcinolone with a custom-made needle was explained by declaring that this procedure may cause an elevation of intraocular pressure or/and form cataract, and informed consent from her parents was taken.

The next day, the suprachoroidal injection was done without any complications. After 1 week, the patient’s BCVA was 0.7, and the OCT image showed a signal of reappearance in the ellipsoid zone (Fig. 1C). After 12 weeks, the patient's BCVA was 0.8. OCT imaging presented a resolution of the overlying increased reflectivity, with the presence of the ellipsoid zone integrity and persistent discontinuity of interdigitation zones (Fig. 1F). Four months of follow-up showed full recovery in the BCVA (1.0) and the retina (Fig. 1G).

During 12 weeks of follow-up, there were no vision-threatening complications. However, intraocular pressure (IOP) increased to 28 mmHg in the seventh week, and was controlled by topical eye drops (timolol) to be 15 mmHg.

**Fig. 2:**
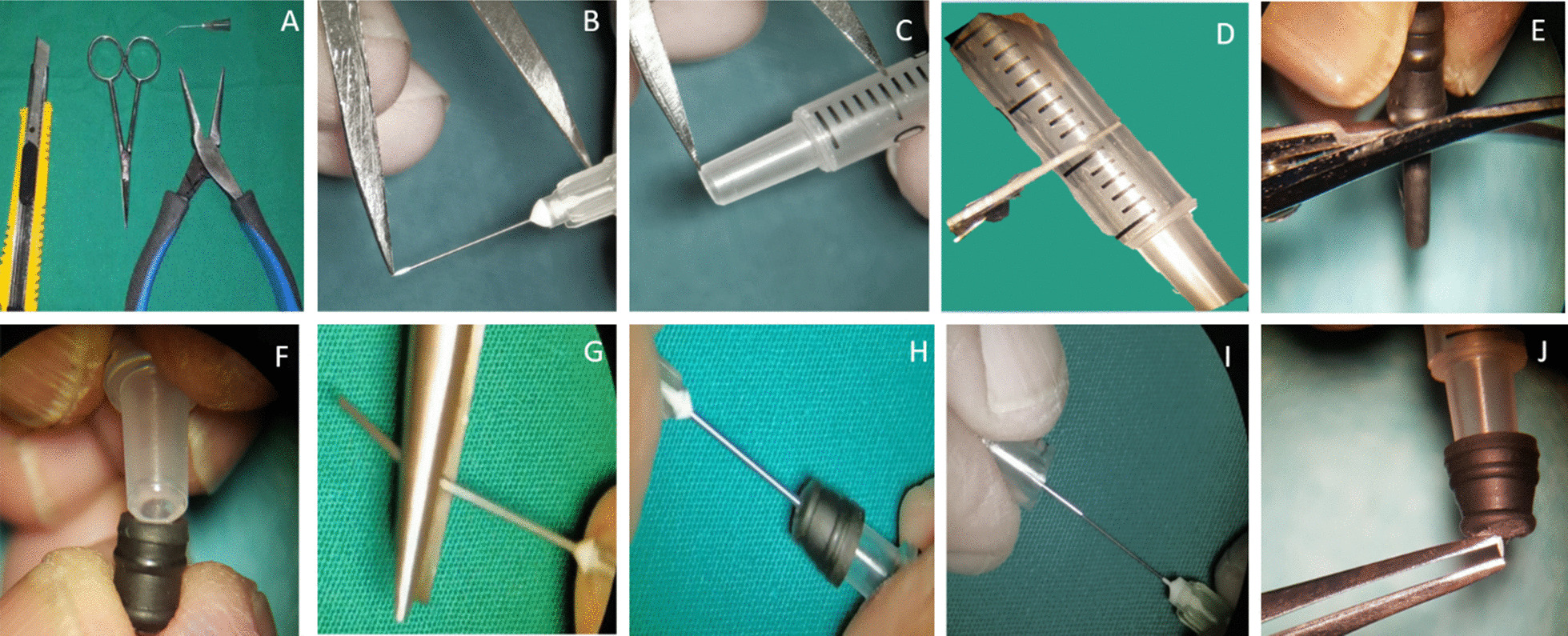
**A** The tools are scissors, calipers, blade, Luer slip syringe, 30-gauge needle, irrigating cannula, and needle-nose pliers. **B** Measuring the needle’s full length, including the part embedded in the needle’s plastic. **C** Preparing the plastic stopper by measuring 2.5 mm (less than 1 mm for the needle, and 1.5 mm was added because of the additional thickness of the rubber seal). **D** Cutting the syringe with a blade according to the measurements. **E** Preparing the stopper’s rubber part by using scissors to remove the rubber seal’s ramifications. **F** Installing the rubber on the prepared plastic Luer slip. **G** Using the needle nose to straighten the irrigating cannula. **H** Implanting the irrigating cannula in the rubber seal through the rubber ring. **I** Using the irrigating cannula as a guide to putting the 30-gauge needle out of the rubber seal. **J** Fixing the plastic Luer slip to the plastic part of the 30-gauge needle.

The tools needed to make this injection manually are scissors, calipers, blade, Luer slip syringe, 30-gauge needle, irrigating cannula, and needle-nose pliers (Fig. 2A). First, we measured the needle’s full length, including the part embedded in the plastic; (Fig. 2B). Then, we prepared the plastic stopper by measuring 2.5 mm (less than 1 mm for the needle, and 1.5 mm was added) (Fig. 2C). First, however, the blade was used to cut the syringe according to the measurements (Fig. 2D). After that, we prepared the stopper’s rubber part by using scissors to remove the rubber seal’s ramifications (Fig. 2E). Next, the rubber stopper was installed on the prepared plastic Luer slip, which will permit only 1000 microns from the 30-gauge needle to penetrate the sclera (Fig. 2F). Next, the needle-nose pliers were used to straighten the irrigating cannula (Fig. 2G), and they were impaled in the rubber seal through the rubber ring (Fig. 2H) and used as a guide to putting the 30-gauge needle out of the rubber seal (Fig. 2I). Finally, the plastic Luer slip was fixed to the plastic part of the 30-gauge needle (Fig. 2J).

After sterilizing the epidermis around the eye, we administered the suprachoroidal injection in sterile conditions, including eyelids and lashes.

**Fig. 3:**
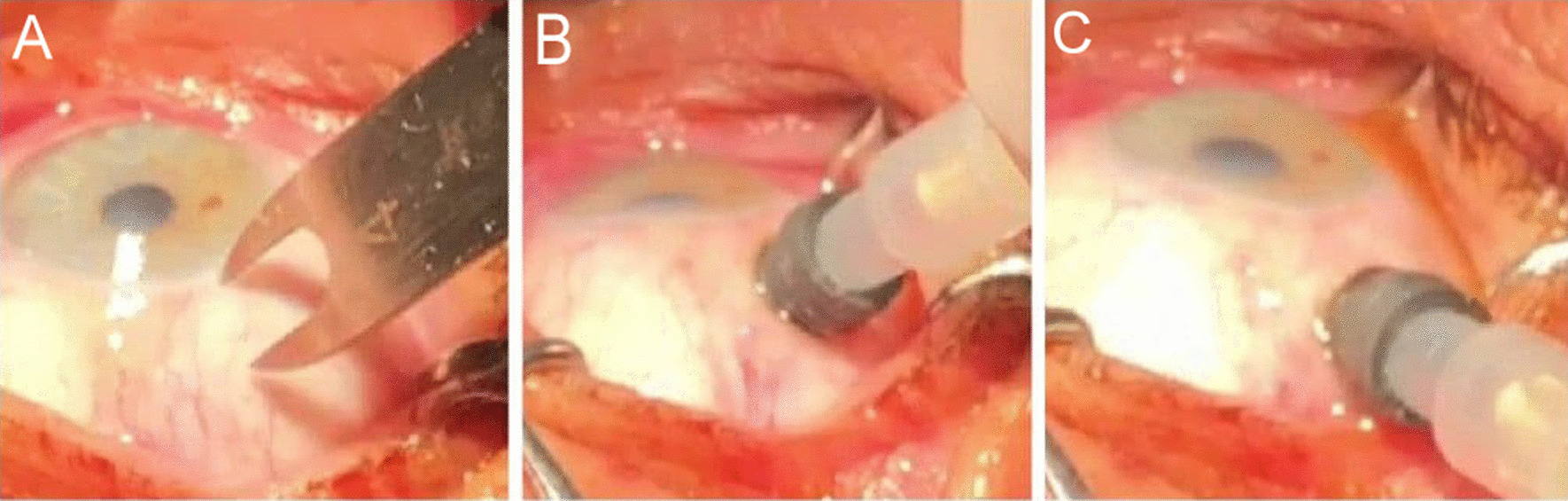
**A** Measuring 4 mm away from the limbus. **B** Insert the needle perpendicular to the sclera and apply gentle pressure on the sclera while injecting. **C** Withdrawing the needle obliquely from the eye.

The injection site was in the temporal quadrant. We measured 4 mm from the limbus using calipers (Fig. 3A) and injected 0.1 ml triamcinolone inside the suprachoroidal space (SCS) (Fig. 3B). The needle was withdrawn obliquely from the eye (Fig. 3C).

## Discussion and conclusion

In this case, solar retinopathy was confirmed with clinical examination and OCT, which showed disruption and discontinuity of the ellipsoid zone, interdigitation zone, and increased reflectivity of the overlying tissue at acute presentation.

Solar retinopathy has been reported as a self-limited disease with recovery within weeks; however, others reported some cases with persistent central scotoma and reduced vision [4, 5]. The variation in outcomes may be due to the difference between levels of damage in retinal tissues and the sun exposure duration [2].

In one study, Atmaca *et al*. evaluated 40 eyes after eclipse retinopathy, and only 35% regained vision of 20/20 spontaneously [6]. In our case, the patient did not spontaneously achieve full visual or anatomical recovery, and the treatment with suprachoroidal triamcinolone was decided.

There is no established treatment for solar retinopathy, and some reports suggested that steroid therapy could suppress the co-existing inflammatory reaction from photopic injury [7, 8].

Nakamura *et al*. reported the improvement of anatomical and functional outcomes after systemic prednisolone and posterior sub-tenon triamcinolone in more severe eye presentation, which suggested the role of steroids in suppressing inflammatory reaction shortening the clinical course in subacute solar retinopathy cases [9].

Our case showed an anatomical improvement, a resolution of overlying retinal tissue reaction, a reappearance of the ellipsoid zone’s signal, a persistent discontinuity of the interdigitation zone, and an improved vision to 1.0 within 4 months post-suprachoroidal injection of triamcinolone.

The purpose of this case report is to draw attention to the clinical efficacy of suprachoroidal triamcinolone injection in subacute solar retinopathy. First, however, a prospective randomized multi-center clinical trial with a larger sample and longer follow-up duration is needed.

## Data Availability

The datasets used and/or analyzed in the current study are available from the corresponding author on reasonable request.

## Abbreviations

SCS: Suprachoroidal space
OCT: Optical coherence tomography
BCVA: Best-corrected visual acuity
RPE: Retinal pigment epithelium
IOP: Intraocular pressure
